# Complex balanced chromosomal translocation t(2;5;13) (p21;p15;q22) in a woman with four reproductive failures

**DOI:** 10.1186/s13039-014-0083-6

**Published:** 2014-11-19

**Authors:** Ewelina Lazarczyk, Malgorzata Drozniewska, Magdalena Pasinska, Beata Stasiewicz-Jarocka, Alina T Midro, Olga Haus

**Affiliations:** Department of Clinical Genetics, Collegium Medicum, Nicolaus Copernicus University, Sklodowskiej-Curie 9, Bydgoszcz, 85-094 Poland; West Midlands Regional Genetics Laboratories, Birmingham Women’s Hospital NHS Trust, Edgbaston, Birmingham, B15 2TG UK; Department of Genetics, Medical University, Waszyngtona 13, Bialystok, 15-089 Poland; Department of Hematology, Blood Malignancies and Bone Marrow Transplantation, University of Medicine, Pasteura 4, Wroclaw, 52-367 Poland

**Keywords:** Balanced complex translocation (BCT), Complex chromosome rearrangement (CCR), Reciprocal chromosomal translocation (RCT), Reproductive failure, Conventional cytogenetics (CC), Fluorescence *in situ* hybridization (FISH)

## Abstract

**Background:**

Balanced complex translocations (BCTs) are rare events, they may result in reproductive failures: spontaneous abortions, missed abortions, stillbirths, congenital malformations in children, and male infertility. BCTs belong to the group of complex chromosome rearrangements (CCRs) – up to date about 260 cases were described.

**Results:**

The described patient and her husband were referred to genetic counseling clinic because of four reproductive failures. GTG-banded chromosome analysis revealed presence of apparently balanced complex translocation t(2;5;13), which was verified and confirmed by molecular cytogenetics with single copy probes. This complex aberration was most likely responsible for reproductive failures in our patient. Since no high resolution molecular karyotyping (microarrays) was used, this rearrangement can only be considered to be balanced at cytogenetic level.

**Discussion:**

Due to small number of reported cases of CCRs/BCTs and individual as well as unique character of such rearrangements, genetic counseling for CCRs carriers is complex and requires detailed pedigree analysis, as well as extended clinical and genetic testing.

## Background

Reciprocal chromosomal translocations (RCTs) are structural aberrations which occur as a result of exchange of chromosome fragments, usually between two nonhomologous chromosomes. When the amount of genetic component is balanced the aberration usually has no influence on patient’s phenotype [1]. Balanced complex translocation (BCT) occurs when more than two chromosomes are involved in the translocation [2,3]. BCTs belong to the group of complex chromosome rearrangements (CCRs) [4]. In general population BCTs occur rarely, that is why every new described case can bring more information on possible consequences of carrying this rearrangement [3,5]. About 260 cases of CCRs have been reported up to date [6-10]. In most of the carriers of such complex translocations, reproductive failures, including spontaneous abortions, stillbirths, delivering children with congenital malformations, and male infertility were present [1,3,4,6,11-13].

There are several different definitions and classifications of CCRs used in the literature, most of which base on the number of chromosomes and the number of breaks involved. Most of them originate *de novo*, however they can be also hereditary, in both balanced and unbalanced forms. The carrier status is typically revealed due to pregnancy failures. In 2012 Madan divided CCRs into four groups [14]. In the type I of CCRs number of chromosomal breaks equals number of chromosomes involved in an aberration and the exchange can be three- or four-directional. In the type II number of breaks is one more than number of involved chromosomes; this type also contains inversion. In the type III, number of breaks is greater than number of involved chromosomes, with the presence of at least one insertion. In the type IV, apart from number of breaks greater than number of chromosomes the occurrence of ‘middle segment’ is observed. ‘Middle segment’ means fragment of a chromosome located in the middle of derivative chromosome, flanked bilaterally by fragments of different chromosomes. In this type of CCRs at least one of derivative chromosomes is composed of three different chromosomes. This type also includes more complex rearrangements, with multiple breakpoints and more than one ‘middle segment’ [14]. In 2013 Madan proposed a new approach to CCRs’ classification. She concluded that in *de novo* cases in phenotypically abnormal individuals the significance of the detected imbalance and its phenotypic effect should be emphasized. However in familial cases it is still important to describe number of chromosomes and breaks involved [8].

Most of the cases of CCRs occur de novo (~70%) and the remaining ones are usually transmitted by mothers [7].

## Case presentation

25-year old woman and her husband were referred to clinical genetics unit due to four pregnancy failures. First pregnancy (anencephalic) was terminated at 19^th^ week of gestation. At 6^th^ week of the second pregnancy blighted ovum was found. Third pregnancy underwent spontaneous abortion at 6^th^ week of gestation. The fourth pregnancy was extrauterine.

The physical examination did not reveal any phenotypic abnormalities or any congenital malformations in either partner.

**Figure 1:**
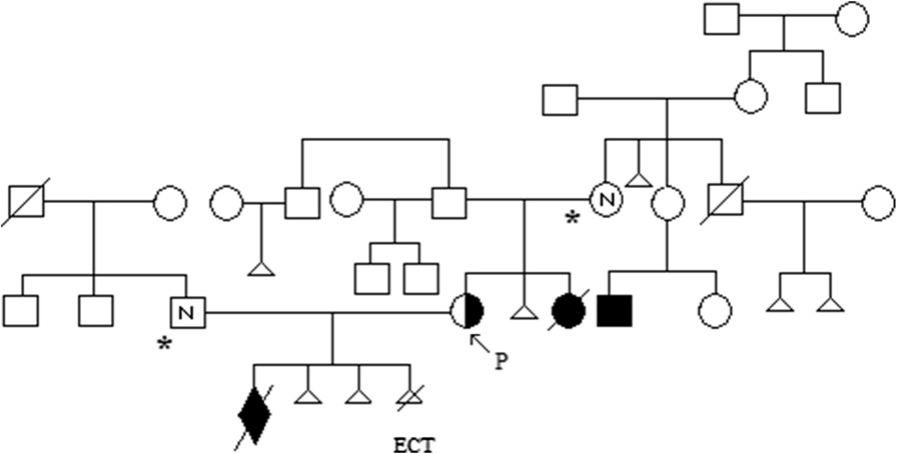
**Patient’s pedigree.** Arrow indicates proband. Proband’s mother and husband are indicated by an asterisk. Only these family members were tested. ‘N’ means normal karyotypes. Only in proband both cytogenetic and FISH testing were performed.

A history of reproductive problems was reported in maternal family members (see Figure 1 showing pedigree). Our patient’s mother had difficulties to conceive, her second pregnancy was spontaneously aborted. She also gave birth to a girl who died before the age of one month due to congenital malformations. The only sister of patient’s mother has one son with heart defect and one healthy daughter. The wife of the brother of patient’s mother had two spontaneous abortions. Maternal grandmother had one spontaneous abortion.

Patient’s father has two healthy sons from his second relationship. Wife of father’s brother underwent a spontaneous abortion of her only pregnancy. Paternal history was otherwise unremarkable.

Family of patient’s husband did not have any history of reproductive health problems.

## Results

**Figure 2:**
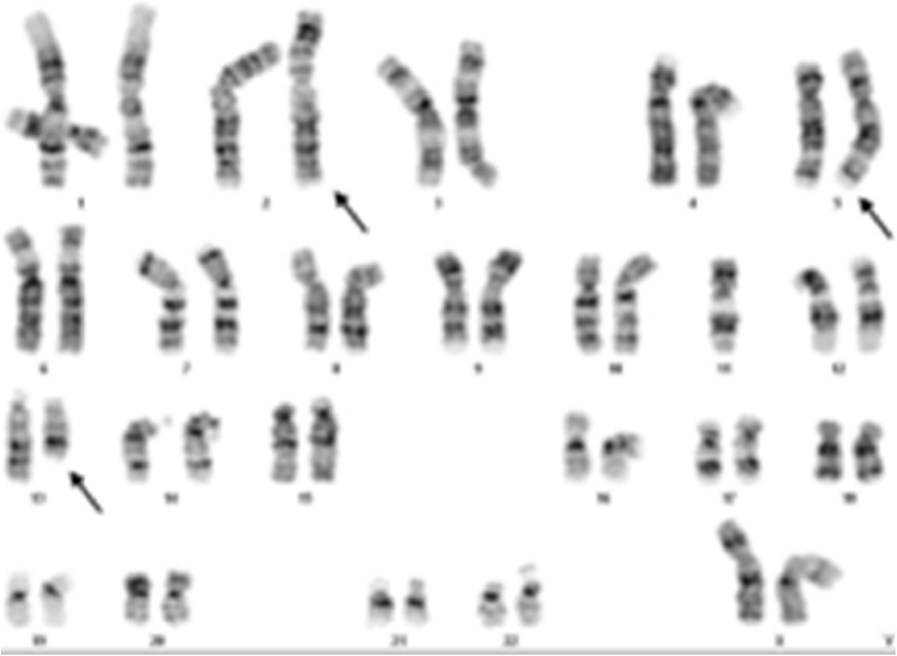
**Karyogram of the patient in GTG-banding showing t(2;5;13) (p21;p15.1;q22).** Arrows indicate abnormal chromosomes.

Classical cytogenetic examination revealed translocation involving chromosomes 2, 5 and 13. Karyotype of the patient was established as 46, XX, t(2;5;13) (p21;p15.1;q22) (Figure 2). Karyotypes of patient’s husband and mother were normal (data not shown).

FISH technique with whole chromosome painting (wcp) probes: wcp2, wcp5, wcp13, and specific probes: D13S1825, *N-MYC*, *DLEU1*, *CTNND2* performed in our patient confirmed the presence of complex translocation involving three chromosomes.

**Figure 3:**
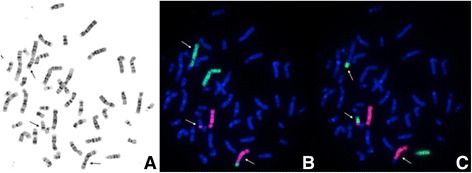
**Combined images of CC and FISH with painting probes. A**. Metaphase spread in GTG-banding obtained from patient’s blood lymphocytes showing t(2;5;13) (p21;p15.1;q22). Arrows show abnormal chromosomes. **B**. The same metaphase as in Figure 3A in FISH technique with painting probes: chromosome 2-green, 5-red. Material from der(2) is present on der(5) while material from der(5) is present on der(13). Arrows show abnormal chromosomes. **C**. The same metaphase as in Figure 3A and 3B in FISH technique with painting probes: 13-green, 5-red. Material from der(13) is present on der(2), while material from der(5) on der(13). Arrows show abnormal chromosomes.

**Figure 4:**
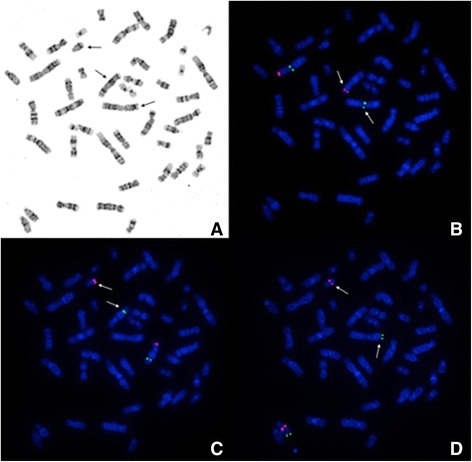
**Combined images of CC and FISH with single copy probes. A**. Metaphase spread in GTG banding showing t(2;5;13) (p21;p15.1;q22). Arrows show abnormal chromosomes. **B**. The same metaphase as 4A with *N-MYC* (2p24) probe in red (control gene – *LAF* (2q11) – green). One of *N-MYC* signals is present on der(5). Arrows show abnormal chromosomes 2 and 5. **C**. The same metaphase as 4A and 4B, with cri-du-chat critical region probe – *CTNND2* (5p15.2) – red. Control region, 5q13 – green. One of the *CTNND2* signals is visible on der(13). Arrows show abnormal chromosomes 5 and 13. **D**. The same metaphase as 4A, 4B and 4C, with *DLEU1* probe (13q14.3) – red. Control region, 13qter, is green. Both signals are present on normal 13 chromosome. The second *DLEU1* signal is present on der(13), and the second control signal on der(2). Arrows show abnormal chromosomes 2 and 13.

Combined GTG-banded metaphase spreads and FISH images illustrating the complex character of this rearrangement are presented in Figures 3 and 4.

Probability of unbalanced karyotype in a child was estimated as 2% (low risk) to 13% (high risk), depending on the type of imbalance. Risk of miscarriages was estimated at around 30%.

No other genetic testing was performed due to the lack of microarray technology in our laboratory.

## Discussion

The aberration found in our patient was most likely responsible for her reproductive health problems. Literature data indicate that the risk of spontaneous abortions in BCT carriers is higher than in carriers of RCT [2-4]. Presence of abnormal phenotypic features could be associated with microdeletions or microduplications accompanying BCT or could be a position effect of genes located at or flanking the breakpoints involved in aberrations [15].

Gorski et al. estimated the risk of spontaneous abortions in BCT carriers at 48.3% and the risk of child malformations at 18.4% [16]. These data are cited by most authors, however it must be stressed that each case of CCR should be considered separately and should require individual approach at genetic counseling due to the lack of reproducibility in general population.

In carriers of CCRs more complex mechanisms of chromosome segregation occur in comparison to translocations involving two chromosomes [2]. Among these mechanisms non-allelic homologous recombination (NAHR) is widely proposed. Alternative mechanisms include non-homologous end-joining (NHEJ) or microhomology-mediated break-induced replication (MMBIR). It has also been proposed that a molecular mechanism similar to chromothripsis (occurrence of different rearrangements in a single chain chromosome breakage event) can be involved [17].

Translocation described in our patient belongs to three-way, three breakpoints exchange CCR, with one breakpoint on each involved chromosome. In 80% of cases from this group, the most expected type of segregation is 3:3, which can determine the formation of 20 types of gametes: 2 balanced and 18 unbalanced. According to the literature, 4:2 segregation is also possible in about 20% of cases [4].

To our knowledge, only one case involving the same chromosomes as seen in our patient (but with different breakpoints – 2q14.2, 5q22-q23.2, and 13q34) has been described so far [18].

**Figure 5:**
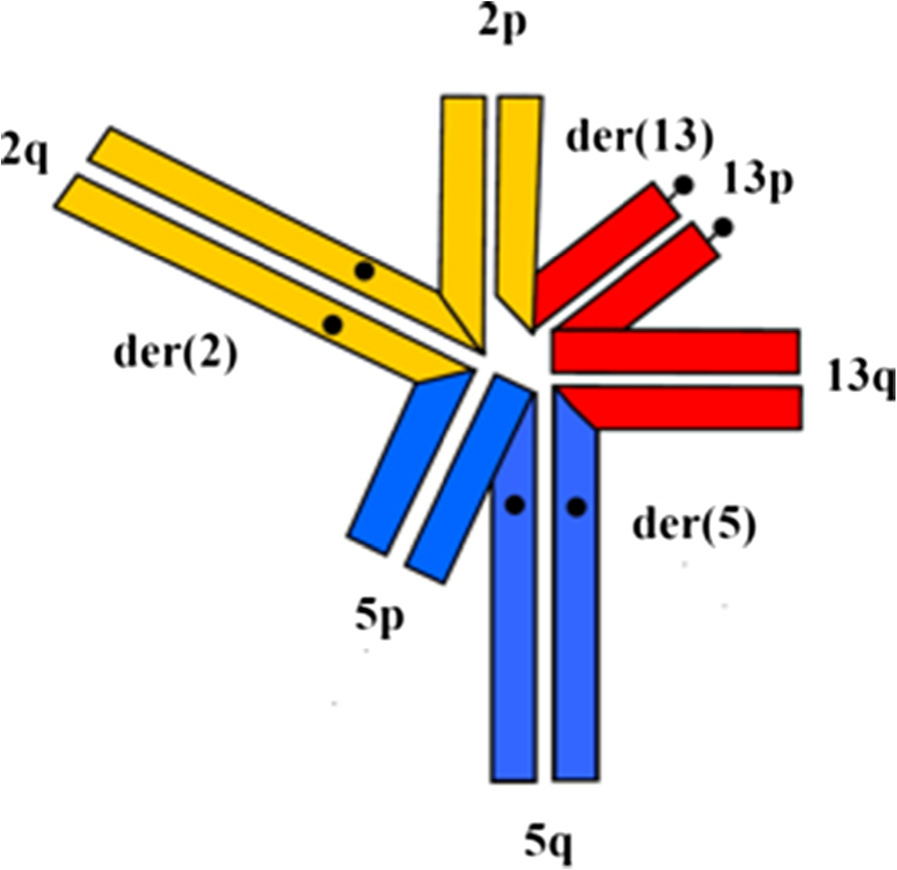
**Schematic representation of hexavalent of meiotic chromosomes involved in t(2;5;13) (p21;p15.1;q22).**

Most of the cases of CCRs are unique, ‘private’ for their carriers or carriers’ families. They are also very rare events, with frequency estimated around 0.1% (frequency of couples with recurrent spontaneous abortion in which one partner carries a balanced translocation between three chromosomes) [19]. The possibility of chromosomally normal or balanced gametes is considered to be low, which can be calculated from the theoretical hexavalent configuration during meiotic cell division (Figure 5).

This complex structural rearrangement can result in partial monosomies or trisomies of involved chromosomes. Not surprisingly, they can result in variable phenotypes. In patients with familial form of partial trisomy of 2p, neural tube disorders were present, including anencephaly, occipital encephalocele or spina bifida [20].

Partial monosomies of 2p are rare. Microcephaly was noted when deletion spanned 2p23-pter region [21]. Microdeletion of 2p15-p16.1 was reported in patient with cerebellar hypoplasia, intellectual disability, microcephaly, optic nerve hypoplasia and autistic behaviour [22]. General symptoms present in patients with partial deletions of short arm of chromosome 2 include developmental delay, growth retardation, feeding difficulties, axial hypotonia, limbs spasticity and spine anomalies [23].

Clinical result of partial deletion of short arm of chromosome 5 may be cat cry syndrome (cri du chat). The most significant symptoms of this syndrome include characteristic cry of the newborn (cat-like cry), facial dysmorphy, microcephaly, severe or profound developmental delay and intellectual disability. Low birth weight, hypotonia, hypertelorism and epicanthal fold are listed as additional features of this syndrome. The ‘final’ phenotype of this syndrome usually depends on the size of deleted region – there are patients in whom cat-like cry is the only feature present [24].

Phenotype of patients with 5p trisomy can be highly variable (cytogenetically and molecularly) due to different duplicated regions of 5p. The most frequent 5p duplications encompass 5p13-pter region and are associated with intellectual disability, dolichocephaly, facial dysmorphism, high arched palate, tongue hypertrophy and micrognathia. Partial trisomy 5p is usually the result of inheritance from a parental derivative chromosome, which results from parental balanced reciprocal translocation or parental inversion, less frequently it results from a insertion or a marker chromosome [24].

Trisomy of 13q is more frequently a result of parental balanced translocation or pericentric inversion rather than of *de novo* duplication [25]. Most of the features present in patients with partial trisomy 13q are also present in patients with full chromosome 13 trisomy which results in Patau syndrome (PS). However, clinical features of full trisomy 13 are more severe due to presence of brain malformations, cardiovascular defects and renal anomalies. In about 80% of PS patients holoprosencephaly with characteristic dysmorphy of midface (e.g. proboscis), cleft lip and palate, small dysplastic earlobes, microcephaly, and hypotelorism are also present [25,26]. In patients with partial 13q trisomy holoprosencephaly occurs rarely, most often when trisomic region includes 13q11-q14 [26].

In partial 13q monosomy phenotypes vary in regard to size and location of a deleted fragment. Severe mental retardation, growth retardation, microcephaly, micrognathia, microphthalmia, cleft palate, absent thumbs, and hypoplastic kidneys are the phenotypic features of this aberration. Postaxial polydactyly is associated to loss of 13q21-q32 region [27].

## Conclusions

The genetic risk of having children with congenital anomalies and the risk of pregnancy losses is in our patient at the high level (2-13% and 30%, respectively). This results from the complexity of possible combinations of chromosome losses and gains. She has a chance of having healthy child, because only one homolog of each chromosome pairs 3, 5 and 13 is involved in the translocation.

It can be stated on cytogenetic/FISH level only that the aberration present in our patient is balanced. It is crucial to characterise and analyse the breakpoints with greater details as some congenital malformations may arise due to a disruption of key genes involved in development of pregnancy.

It is difficult to predict the likely phenotypic outcome of any future pregnancies or children of described patient, as many different forms of chromosome imbalances may occur in her gametes. Thus, genetic counseling may be very difficult and complex. The patient should be offered invasive prenatal diagnosis in future pregnancies.

As the presence of any chromosomal rearrangement was excluded in the patient’s mother by standard cytogenetic analysis, reproductive failures in members of maternal line are not relevant to this case. They, most likely coincide with the carrying of t(2;5;13) by the patient.

The origin of the translocation, paternal or de novo, could not be established due to the lack of consent of patient’s father for the cytogenetic examination.

Despite the wide usefulness of microarray technology in detecting genome imbalances in apparently balanced chromosomal rearrangements, some laboratories still have no access to this technology. The authors will continue to investigate this case using array CGH technique.

This case does not provide any major breakthrough, however we strongly believe that it is still worth to publish every case of CCR due to its unique character as it has been proposed by Guilherme et al.: ‘a better characterization of the CCRs is important for a better knowledge of their mechanisms of formation and their relevance to phenotype’ [17].

## Materials and methods

5 ml of peripheral blood was taken from each: the patient, her husband and mother. The patient’s father did not give his consent for blood sample. Blood cells were cultured according to standard procedures. Cytogenetic slides were stained with GTG banding technique and described according to ISCN 2013.

GTG-banded chromosomes analysis revealed translocation involving chromosomes 2, 5 and 13. In order to confirm the three-way character of this abnormality, fluorescence *in situ* hybridization (FISH) was performed. The following molecular probes were used: whole chromosome painting probes (wcp) for chromosomes 2, 5 and 13 (Cytocell, UK), and specific probes – D13S1825 (Cytocell, UK), *N-MYC* (2p24) (Kreatech Diagnostics, Holland), critical region for cri-du-chat *CTNND2* (5p15.2) (Kreatech Diagnostics, Holland) and *DLEU1* (13q14.3) (Cytocell, UK). FISH analyses were performed according to manufacturers’ procedures. Images were analysed with Spectral Imaging system with FISH module (Applied Spectral Imaging, USA).

## Consent

Written informed consent was obtained from the patient for publication of this case report and any accompanying images. A copy of the written consent is available for review by the Editor-in-Chief of this journal.
